# Ten common issues with reference sequence databases and how to mitigate them

**DOI:** 10.3389/fbinf.2024.1278228

**Published:** 2024-03-15

**Authors:** Samuel D. Chorlton

**Affiliations:** BugSeq Bioinformatics Inc., Vancouver, BC, Canada

**Keywords:** metagenomic, reference, sequence, database, taxonomy

## Abstract

Metagenomic sequencing has revolutionized our understanding of microbiology. While metagenomic tools and approaches have been extensively evaluated and benchmarked, far less attention has been given to the reference sequence database used in metagenomic classification. Issues with reference sequence databases are pervasive. Database contamination is the most recognized issue in the literature; however, it remains relatively unmitigated in most analyses. Other common issues with reference sequence databases include taxonomic errors, inappropriate inclusion and exclusion criteria, and sequence content errors. This review covers ten common issues with reference sequence databases and the potential downstream consequences of these issues. Mitigation measures are discussed for each issue, including bioinformatic tools and database curation strategies. Together, these strategies present a path towards more accurate, reproducible and translatable metagenomic sequencing.

## 1 Introduction

Metagenomic sequencing has unlocked the ability to rapidly study and understand the content and role of microbes in clinical, environmental, industrial and research applications. It has enabled the diagnosis of disease, identification of pandemic agents and revealed the microbial importance of our microbiome and environment (Tringe and Rubin, 2005; Gilbert et al., 2018; Chiu and Miller, 2019; Huang et al., 2020). After random sequencing of DNA or RNA, metagenomic analysis typically involves comparison of sequenced reads or assembled contigs against a reference database for taxonomic classification. While the bioinformatic methods, including quality control, preprocessing and method of classification are vital to translating this data into knowledge and understanding, the reference sequence database serves as a ground truth for comparison and is of paramount importance. Changing the reference sequence database can lead to significant changes in the accuracy of taxonomic classifiers, and therefore the understanding derived from analysis (Ye et al., 2019; Wright et al., 2023). In a sensationalist example, Marcelino, Holmes and Sorrell demonstrated the detection of turtles, bull frogs and snakes in human gut samples by changing the reference sequence database of a published analysis (Marcelino V et al., 2020). More practically, changes to the reference sequence database affect the number of reads classified, the recall and precision of taxa, computational efficiency of classification, and diversity and distance metrics (Méric et al., 2019; Gihawi et al., 2023; Wright et al., 2023).

It is well-established that issues with reference sequences databases are pervasive. Contamination is probably the most recognized database issue; systematic evaluations have identified 2,161,746 contaminated sequences in NCBI GenBank and 114,035 contaminated sequences in RefSeq, its higher quality subset (O Leary et al., 2016; Steinegger and Salzberg, 2020). Others have reviewed causes and mitigation strategies for sequence contamination (Cornet and Baurain, 2022). However, database issues go well beyond contamination, and default databases used in the most popular tools are affected by taxonomic errors, inappropriate inclusion and exclusion criteria, and errors with sequences themselves. This phenomenon is a result of most metagenomic tools simply mirroring NCBI resources, including NCBI GenBank, RefSeq, Taxonomy, and BLAST nucleotide database (nt), into their database (Kim et al., 2016; O Leary et al., 2016; Breitwieser et al., 2018; Wood et al., 2019; Piro et al., 2020; Schoch et al., 2020; Blanco-Míguez et al., 2023).

These resources have undergone important and extensive curation by the NCBI team, which is reviewed elsewhere (O Leary et al., 2016; Schoch et al., 2020). They are also the only resources, to date, which encompass all taxonomic kingdoms. We therefore choose to focus on them in this review. Genome Taxonomy Database (GTDB) and MetaPhlAn have made efforts to curate reference sequence databases; however, their curation is almost entirely limited to prokaryotes, precluding vital understanding of most ecological niches which extend to viruses, fungi and other eukaryotes (Parks et al., 2022; Blanco-Míguez et al., 2023). Furthermore, applications of the GTDB and MetaPhlAn databases are limited, as these resources do not follow medically accepted taxonomy. For example, both GTDB and MetaPhlAn collapses similar species such as *Escherichia coli* and *Shigella* spp. into a single taxon (Parks et al., 2020; Blanco-Míguez et al., 2023).

A comprehensive list of database issues, along with examples and mitigation strategies, has yet to be compiled. While not exhaustive, this review covers ten common issues with reference sequence databases and how to mitigate them (Table 1). Issues which are prevalent and problematic, yet beyond the scope of this review, include synonymous taxonomic names, protein database curation, database versioning and provenance, and regulatory compliance. This review also does not cover curation of antimicrobial resistance, plasmid sequence and strain typing (e.g., core genome multilocus sequence typing) databases, which in our experience, are even more technically complex and plagued with challenges compared to reference sequence databases.

**TABLE 1:** Summary of popular issues with reference sequence databases and mitigation strategies.

Issue	Mitigation strategies
1. Incorrect taxonomic labelling	• Comparison of sequences against type material
	• Extensive database testing and use
2. Unspecific taxonomic labelling	• Review of label distribution across ranks
	• Identification of unspecific taxon names, such as those including “sp.”
3. Taxonomic underrepresentation	• Broad database inclusion criteria
	• Sourcing of sequences from multiple repositories to fill underrepresented taxa
4. Taxonomic overrepresentation	• Selective inclusion criteria
	• Sequence deduplication or clustering
5. Inappropriate inclusion or exclusion of host, vectors and non-microbial taxa from database	• Inclusion of best available host reference genome
	• Intentional inclusion and exclusion of taxa tailored for the ecological niche under study
	• Inclusion of common vector and contaminating sequences
6. Partitioned sequence contamination	• Assessment of sequences with tools such as BUSCO, CheckM, EukCC, compleasm and others (Parks et al., 2015; Saary et al., 2020; Manni et al., 2021; Huang and Li, 2023)
7. Chimeric sequence contamination	• Assessment of sequences with tools such as GUNC, CheckV, Kraken2 or Conterminator (Wood et al., 2019; Steinegger and Salzberg, 2020; Nayfach et al., 2021; Orakov et al., 2021)
8. Poor quality reference sequences	• Strict quality control of included sequences for fragmentation, completeness, circularity and other measures
9. Low complexity masking	• Masking of low complexity sequences if compatible with the classification algorithm
10. Database maintenance and updating	• Team approach to database management
	• Dedicated resources for database curation and updating
	• Automation of quality control procedures

## 2 Issues and mitigation strategies

### 2.1 Incorrect taxonomic labelling

Taxonomic misannotation, or simply, assigning the incorrect taxonomic identity to a sequence, is a common cause of database error. Misannotation may result in false positive taxa detections, false negative detections or imprecise (but technically correct) classifications. Misannotation is usually the result of data entry error or incorrect identification of the sequenced material by a data submitter (Federhen, 2015). Partly, this is a consequence of the lack of a standard for the taxonomic annotation of sequences by a submitter, or may also result from failure of the submitter to adhere to existing standards (Table 2). While conventional techniques, such as matrix-assisted laser desorption/ionization mass spectrometry (MALDI-TOF MS), biochemical tests and 16S rRNA gene sequencing are generally accurate for bacterial identification, they are susceptible to error (Janda and Abbott, 2007; Patel, 2015). For example, MALDI-TOF MS and 16S rRNA gene sequencing cannot reliably differentiate closely related organisms such as *E. coli* and *Shigella* species, which have near-identical 16S rRNA gene sequences (Patel, 2015; Devanga Ragupathi et al., 2018). Compounding issues with 16S rRNA gene sequencing is the fact that it only reflects a single locus of a prokaryotic genome and does not encapsulate the complexities of the core and accessory genome, including hundreds to thousands of genes which undergo evolution and horizontal gene transfer. Similar to Issue #3 in this review, older systems such 16S rRNA gene sequencing and MALDI-TOF may misclassify organisms if the true identity is missing from the reference database (Patel, 2015). Fungal, viral and parasitic identification may be even more complicated, with many laboratories still relying on microscopy or nucleic acid amplification for identification. Novel and emerging organisms are also particularly prone to misidentification.

**TABLE 2:** Example standards for taxonomic curation and assignment. Examples encompass a mix of kingdom- and clade-specific standards.

Taxonomic group	Example standard
All	Stewart et al. (2018), Yu et al. (2023)
Bacteria and Archaea	Bowers et al. (2017)
Viruses	Kuhn et al. (2014), Ladner et al. (2014), Roux et al. (2019)
Fungi	Lücking et al. (2021)
Symbiont-associated micro-organisms	Jorge et al. (2022)

Taxonomic misannotation is pervasive in NCBI GenBank and RefSeq: it has been estimated to affect 3.6% of prokaryotic genomes in GenBank and approximately 1% of its curated subset RefSeq (Ciufo et al., 2018; Lupo et al., 2021). In 2018, NCBI reported that they were flagging 75 genome submissions per month for review based on unexpected taxonomic annotation, and that number has likely only grown since (Ciufo et al., 2018). While NCBI performs review of submitted data for accurate taxonomic classification, GenBank records are owned by the data submitter and cannot be modified by NCBI. Furthermore, NCBI may not have necessary data available to them to confirm taxonomy, with a reported 137,000 GenBank records unable to be validated due to insufficient data (Kannan et al., 2023). Certain taxonomic branches may be more affected by error, with up to 35.9% taxonomic discordance reported for the *Aeromonas* genus (Beaz-Hidalgo et al., 2015). Many individual cases of misannotation are reported in the literature as well. For example, NCBI assembly GCF_900453015.1 was originally misidentified as *Micrococcus lylae*, with its identity since updated to *Macrococcus caseolyticus* and two *Raoultella ornithinolytica* assemblies (GCA_000703465.1 and GCA_000703485.1) were originally submitted as *E. coli* (Federhen, 2015; Lupo et al., 2021). A comprehensive list of misannotations is beyond the scope of this review.

Misannotated sequences can be systematically detected and either corrected or excluded from a reference sequence database. Detection of misannotated sequences can be accomplished by comparing sequences against a known gold-standard or other sequences in the database (Beaz-Hidalgo et al., 2015; Ciufo et al., 2018; Kannan et al., 2023). For example, comparing CP001654, submitted as *Dickeya dadantii*, to type material (trusted material deposited in at least two culture collections), enabled NCBI to correct its annotation to *Dickeya paradisiaca* (Federhen, 2015)*.* Generally, species follow a 95%–96% Average Nucleotide Identity (ANI) demarcation enabling outliers to be identified and reviewed (Ciufo et al., 2018). Not all species follow this rule, such as *E. coli* and *Shigella* spp, which maintain legacy exceptions, and therefore care must be taken when clustering sequences by ANI.

Others, such as the FDA-ARGOS project, have suggested a more restrictive approach where only sequences with a robustly verified identity are included in the database (Sichtig et al., 2019). While technically appealing, this approach is practically onerous and has resulted in databases with significant taxonomic underrepresentation (Issue #3), precluding their widespread adoption (Gauthier et al., 2023). Finally, a database testing process can detect taxonomic misannotation. Processing thousands or more of diverse samples frequently reveals false positive detections which can be investigated further. Reference sequence databases for use in critical applications, such as clinical metagenomics, should be validated across thousands of samples and manually curated to ensure appropriate detection and correction of edge cases.

### 2.2 Unspecific taxonomic labelling

Occasionally, sequences are technically annotated to an accurate taxon but not annotated to the most specific leaf in the taxonomic tree. As an extreme example, annotation of all bacterial sequences to the NCBI taxonomic node “Bacteria” would preclude speciation of bacteria in a sample. The currently accepted NCBI taxonomy includes deep branching taxonomies to the strain, subtype and serotype rank; reference sequences should be annotated to the deepest node in the taxonomic tree while maintaining accuracy. Analysis tools may then leverage sequence homology, coverage and other factors to determine the most confident or accurate taxonomic classification of a query sequence (Chandrakumar et al., 2022).

A real example of unspecific taxonomic labelling causing imprecise metagenomic analysis is Respiratory Syncytial Virus (RSV). RSV subgrouping has clinical and public health implications, and assigning reads to a single RSV subgroup via metagenomic classifier should be feasible based on the relatively low absolute nucleotide identity shared between the two subgroups and absence of subgroup recombination (Yu et al., 2021). However, public sequence annotation precludes this analysis because both subgroup A and B RefSeq sequences are annotated to the Human orthopneumovirus taxon (ID 11250), despite there being more specific taxonomic nodes in NCBI’s taxonomy (Muñoz-Escalante et al., 2019; Ramaekers et al., 2020).

Another example of unspecific taxonomic labelling is SARS-CoV-2. In NCBI’s taxonomy, SARS-CoV-2 is a child of viral species SARS-CoV with rank “no rank.” However, some data submitters have only annotated their submitted SARS-CoV-2 sequences to the parent viral species SARS-CoV. If one includes the NCBI Viral Genome Neighbors in their reference sequence database; as suggested by Breitwieser, Baker and Salzberg; one will also include 37 sequences labelled as SARS-CoV (NCBI taxonomic ID 694009) (Breitwieser et al., 2018). Inspecting these sequences reveals 11 were added to NCBI after 2014 and at least nine of 11 are annotated by the submitter as SARS-CoV-2. Classifying reads against a database with these sequences will result in most SARS-CoV-2 reads being classified to the species rank based on lowest common ancestor. While technically accurate, reporting classification of these reads as SARS-CoV may cause confusion with the eradicated virus from the 2003 SARS-CoV outbreak, and results in imprecise abundance estimation.

Unspecific taxonomic labelling can be systematically corrected with a detailed examination of taxon labelling and distribution in a database. For example, sequences annotated to taxa with high ranks, such as genus, family or above, can be investigated to determine if they can be classified to lower ranks using their annotation or sequence homology and coverage. Occasionally these sequences will be annotated to a specific rank but with an imprecise name; there are 307 sequences in RefSeq as of August, 2023 with taxon label “*Escherichia* sp.,” and therefore keywords must be used to identify these imprecisions. Taxa important for specific applications, such as the aforementioned RSV and SARS-CoV-2 in public health and clinical metagenomics, can be manually corrected with curation.

### 2.3 Taxonomic underrepresentation

Ensuring broad taxonomic representation for the biological specimen and sequencing application is paramount to achieving accurate classification. It is impossible to precisely detect a taxon as present if not represented in the reference database, and reads may match to a near neighbor, giving a false positive classification. Even advanced methods of sequence classification leveraging absolute nucleotide identity or conserved markers may be able to detect an organism without reference sequence but will not be able to identify to the species rank or below (Chandrakumar et al., 2022).

Some popular databases, such as that used in MetaPhlan, do not include all taxonomic kingdoms by design and are therefore not suited to applications such as clinical metagenomics requiring detection of all pathogens, including viruses and eukaryotes (Blanco-Míguez et al., 2023). Outside of these cases, popular taxonomic databases, including RefSeq, vastly underrepresent the taxonomic diversity of many ecological niches. For example, more than 40% and 60% of metagenomic reads from skin and stool, respectively, remained unmapped to any genomic positions when analyzed against a reference database that combined the Human Microbiome Project, and manually selected bacterial, archaeal, and fungal genomes from RefSeq that are present on human skin (Oh et al., 2016). Other studies have supported these estimates: 58 ± 2.2% of human gut species richness was estimated to be uncharacterizable with a different reference database composed of bacterial and archaeal genomes (Sunagawa et al., 2013).

Even if a species is present in a database, underrepresentation of strain diversity may cause missing accessory genes or sequence variation, leading to missed detection. As of July, 2023, there are 35,864 bacterial species in RefSeq represented by only a single genome. Yet, the pangenome of many bacterial species continues to grow after one thousand genomes are sequenced (Park et al., 2019). Furthermore, many databases particularly underrepresent viruses, fungi and parasites, impacting detection and classification accuracy. Breitwieser, Baker and Salzberg found that including NCBI Viral Genome Neighbors led to an increase of up to 20% more reads classified using KrakenUniq in comparison to the standard RefSeq database (Breitwieser et al., 2018). Lu and Salzberg showed that using RefSeq for metagenomic classification would have missed a *Anncaliia algerae* corneal infection which was detected if using VEuPathDB, a more comprehensive database for eukaryotic pathogen genomes (Lu and Salzberg, 2018; Amos et al., 2022).

The reasons for taxonomic underrepresentation (and conversely, overrepresentation, Issue #4) are multifactorial and include both technical and non-technical factors. From a technical perspective, certain organisms or organism groups are harder or more expensive to sequence given the limitations of commercially available technology. In general, longer genomes require more sequencing data to achieve sufficient coverage for assembly, increasing cost. GC content and repetitive elements also make assembly more challenging, while certain organisms cannot be cultured, making isolation of their genomes dependent on metagenomic approaches (Chen et al., 2013; Bowers et al., 2017; Browne et al., 2020). From a non-technical perspective, there is systematic bias for prioritizing and funding sequencing of organisms causing greater human burden of disease or economic impact in higher income populations (Johnson and Parker, 2020; Inzaule et al., 2021; Vries et al., 2021).

Several steps can be taken to ensure broad taxonomic representation in a reference sequence database. First, ensuring broad inclusion criteria may increase the taxonomic representation of a database and improve classification accuracy. Multiple groups have independently shown that the top performer in their reference sequence database evaluations was the largest database evaluated (Méric et al., 2019; Wright et al., 2023). Specifically, Wright et al. found their database with all of NCBI RefSeq and nt, Plasmid and UniVec_Core (“NCBI RefSeq Complete V205”) performed best, while Meric et al. found their database “GTDB_r86_46k” with manual dereplication of GTDB performed best (Méric et al., 2019; Wright et al., 2023). Others have shown that taxon-specific reference databases perform especially poorly (Marcelino V et al., 2020). Taxonomic representation of specific taxonomic groups can be added by sourcing sequences from dedicated or ancillary sources. For example, viral diversity can be increased by include NCBI Viral Genome Neighbors in a database; however, care must be taken as these sequences can also be lower quality or contaminated, as per the issues above. Fungal and parasitic sequences not found in RefSeq may be sourced from the VEuPathDB project; however, these too are also often contaminated and incomplete (Lu and Salzberg, 2018; Amos et al., 2022). Finally, manual curation may be taken to include additional appropriate genomes in a reference sequence database, such as those found in a local outbreak or population.

### 2.4 Taxonomic overrepresentation

In contrast to taxonomic underrepresentation, taxonomic overrepresentation is the inclusion of sequences which do not add value to metagenomic classification. Taxonomic overrepresentation may compromise the efficiency and speed of metagenomic classification or preclude generation of an indexed database (see Issue #10). Twenty pathogenic bacterial species account for more than half of the prokaryotic genomes included in RefSeq (*n* = 54,663/95,336 as of 2017) (Haft et al., 2018). As of July, 2023, this now includes 33,979 *E. coli* genomes, 16,891 *Klebsiella pneumoniae* genomes and 15,497 *Staphylococcus aureus* genomes; Kim et al. showed that reducing redundant sequences for these species resulted in 6.1X, 4.9X, and 7.1X database compression ratios, respectively (Kim et al., 2016).

Paradoxically, taxonomic overrepresentation may also reduce classification accuracy of sequences deriving from overrepresented species. At the most basic level, inclusion of an increasing number of genomes for a taxon makes quality control of those genomes harder: quality control tools may not scale to thousands of genomes, and the included genomes are more likely to contain outlier issues. Even with proper quality control of these genomes, many metagenomic classifiers rely on alignment of sequencing reads against a database, yet many do not appreciate that these sequence aligners filter out the most frequent sequences from their seeding processes, the first stage of alignment, which may impact downstream taxonomic profiling and abundance estimation. As an example, several classifiers are based on minimap2, which as of version 2.26, filters the top 0.02% most frequent minimizers from its index, or SNAP, which as of version 2.0.2 filters single-end read seeds occurring more than 300 times in its index (Fan et al., 2021; Plyusnin et al., 2023; Li, 2018; Naccache et al., 2014; Zaharia et al., 2011). Filtering high frequency seeds may prevent these alignment tools from identifying matches to the overrepresented taxa. Zhou, Gay and Oh demonstrated this with up to a 46.5% increase in the number of reads classified when using a deduplicated sequence database while preserving taxonomic accuracy (Zhou et al., 2018).

Taxonomic overrepresentation can be mitigated with selective inclusion criteria and sequence deduplication. Some have opted to build reference sequence databases by only selecting one or a fixed number of genomes per species (e.g., designated reference or representative genomes), thereby normalizing taxonomic representation (Zhou et al., 2018; Piro et al., 2020). Others have examined including only unique regions of each taxon in a reference sequence database (Kim et al., 2016; Zhou et al., 2018). Others, mainly working with protein, 16S rRNA gene or specialized databases, have clustered sequences to reduce duplication (Quast et al., 2013; Steinegger and Söding, 2018; Blanco-Míguez et al., 2023). Care must be taken with all these approaches to avoid taxonomic underrepresentation (Issue #4); Wright Comeau and Langille found worse metagenomic classification with their non-redundant database compared with their redundant database (Wright et al., 2023). It is likely that future metagenomic classifiers will leverage recent advances in pangenomics and graph-based algorithms to represent sequences not as deduplicated linear sequences but as paths through a graph. Already there is evidence that these approaches can normalize databases by preserving structural and local variation while removing redundancy (Karasikov et al., 2020).

### 2.5 Inappropriate inclusion or exclusion of host, vectors and non-microbial taxa from database

Similar to taxonomic overrepresentation, the inclusion and exclusion criteria for host, vector and non-microbial taxa are paramount to ensuring accurate taxonomic classification. Inclusion of reference sequences which are implausible to find in the sequenced material or ecological niche increases the risk of false positive and false negative classifications. Marcelino, Holmes and Sorrell demonstrated the detection of turtles, bull frogs and snakes in human gut samples using an inappropriate, taxon-specific database (Marcelino V et al., 2020). A more common example of this issue is the use of NCBI nt for metagenomic analysis of human clinical samples with popular pipelines (Naccache et al., 2014; Kalantar et al., 2020). NCBI nt includes diverse taxa, including the 40 Gbp West african lungfish, 32 Gbp axolotl and 20.5 Gbp Sitka spruce genomes, along with more closely related species such as non-human primates. Not only does inclusion of such large genomes make the database unwieldy (Issue #10), it also predisposes to false positives because these genomes are often contaminated (Issues #6 & #7).

For host-associated metagenomic studies, the host species should be included in the reference sequence database. Including the host ensures that host reads are not misclassified. Even with host read removal before metagenomic classification, no approach is perfect and often a metagenomic classifier is used as one step to identify host reads (Bush et al., 2020). The choice of the host reference sequences is paramount. The T2T human genome has been shown to contain nearly 200 million additional base pairs over the GRCh38 genome, including 14.9 Mbp unique sequence when considering 50-mers. These additions result in 0.97% absolute increase in paired reads mapping to the human genome with a 20%–25% reduction in per-read mismatch rate (Aganezov et al., 2022). Others have shown the failing to classify human reads accurately, because of reference genome selection, impacts metagenomic results and conclusions (Gihawi et al., 2023). Despite massive advances in human genomics, the bioinformatics community has been slow to adopt new reference genomes. Even in 2016, 3 years after release of the GRCh38 reference, the 2009 GRCh37 genome accounted for 70% more public BAM submissions to SRA (Schneider et al., 2017). Indeed, metagenomic-specific publications in high-impact journals continue to use GRCh37 as their reference genome into 2023 (Tomofuji et al., 2023). Further care must be taken to choose the T2T assembly with the added Y chromosome, as well as not to use the preformatted NCBI human indices, some of which intentionally contain Epstein Barr Virus and may confound metagenomic analysis if looking for this virus. Databases for use in metatranscriptomic experiments should also include the host’s transcriptome in the database, as splicing may yield additional k-mers or sequences not present in the genome. The choice of host transcriptome source is also important and has been previously reviewed (Zhao and Zhang, 2015).

Other considerations when choosing non-microbial sequences include the choice of vector and artificial sequences. NCBI has published two versions of UniVec, including a full and a core version, which vary on their comprehensiveness and trade recall for precision. Notably, NCBI states that “stretches of sequence [longer than 50 bp] are not necessarily represented as one contiguous piece in UniVec,” meaning that vector sequences may cause false positives if they map to another reference sequence with a longer alignment (Issue #6). EMBL-EBI has also published a contaminant and vector database, emvec, included in some reference sequence databases (Kim et al., 2016; Breitwieser et al., 2018). The emvec database notably contains the human mitochondrial genome, which can lead to unexpected classifications if all sequences in the vector database are uniformly assigned the same taxonomic identity, as is performed by Centrifuge and similar tools (Kim et al., 2016).

### 2.6 Partitioned sequence contamination

Sequence contamination is the most publicized database issue. We use partitioned sequence contamination to refer to the presence of sequences from multiple organisms cleanly divided across different contigs or scaffolds in the same assembly. This issue contrasts with chimeric sequence contamination (Issue #7), where the contamination occurs within the same contig or scaffold. Another scheme to classify sequence contamination is redundant and non-redundant contamination; however, this scheme does not fully capture the different causes and mitigation strategies for each type (Cornet and Baurain, 2022). The reader is directed to Cornet & Baurain for a review of causes of sequencing contamination (Cornet and Baurain, 2022). In brief, partitioned sequence contamination may arise from a contaminated biological specimen, sample preparation (e.g., kit-ome—the microbial content of sequencing reagents) or inaccurate metagenomic binning. An underappreciated reason for sequence contamination is barcode crosstalk, which on some sequencing platforms may affect up to 0.3% of reads (Wick et al., 2018; Xu et al., 2018).

Partitioned sequence contamination is pervasive in reference sequence genomes. For example, of some reports, the cow genome was found to have 173 contaminated contigs of bacterial origin, including *Acinetobacter*, *Pseudomonas* and *Stenotrophomonas* (Merchant et al., 2014). Viral genome contamination was found to comprise 14.5% complete contigs (Chen et al., 2022). GCF_003286725.1, an isolate of *Aerococcus urinae*, is contaminated with *Afipia broomeae* at contig boundaries and at least 5% of cyanobacterial genomes are contaminated (Cornet et al., 2018; Orakov et al., 2021).

Although not exactly contamination, inclusion of plasmid sequences with genome assemblies is another cause for metagenomic misclassification. NCBI assigns plasmid sequences the taxonomic identity of their observed host (Schoch et al., 2020). However, plasmids are often mobile, and observation in a single host does not predict the full host range of the plasmid, which may yet to be sampled (Robertson et al., 2020).

The most popular methods for assessing partitioned sequence contamination in bacteria are CheckM and BUSCO (Parks et al., 2015; Manni et al., 2021). BUSCO, along with other methods such as EukCC and compleasm, can also assess eukaryotic reference sequences (Saary et al., 2020; Manni et al., 2021; Huang and Li, 2023). These tools all function on a similar basis: the conservation of a set of single copy orthologous genes. Detection of duplicate single copy orthologs reflects contamination. Notably, all these approaches only flag contaminated genomes, usually necessitating complete genome removal if not investigated further. If there is only a single reference genome of a species, as is the case for many taxa, this may lead to complete removal of all representation of the organism without further intervention. If raw reads are available for a sample, the genome may be reassembled after filtering contaminating reads, thereby reducing the likelihood of assembling contaminated contigs. Most tools filter contaminating reads by aligning them against a reference genome or using k-mer based classification (Rachtman et al., 2021; Rumbavicius et al., 2023). Contaminated contigs may also be identified and removed using approaches described in Issue #7. Finally, popular approaches such as CheckM and BUSCO only assess for intra-lineage contamination, such as one bacterium contaminating another bacterium. Inter-lineage approaches to contamination detection, such as alignment of contigs across kingdoms, are discussed below.

### 2.7 Chimeric sequence contamination

As opposed to having sequences from multiple organisms occupy different contigs or scaffolds in the same assembly (Issue #6), chimeric sequence contamination is the joining of sequences originating from different organisms into the same contig or scaffold. Chimeric sequence contamination may arise from a contaminated biological specimen or sequencing procedure confounding the assembly process. As early as 1992 it was reported that vector sequences used to clone and sequence samples contaminated 0.23% of sequences in Genbank (Lamperti et al., 1992). Contaminating vector sequences are often found at the ends of contigs, and continue to be submitted to public repositories (Schäffer et al., 2018). Similarly, chimeric sequences of metagenome-assembled genomes may result from assembly errors. Cornet et al. found chimeric contigs in at least 0.5% of cyanobacterial assemblies (Cornet et al., 2018). Steinnegger and Salzberg identified *Acidithiobacillus thiooxidans* contamination of a human reference (GRCh38) scaffold and *E. coli* contamination of the *Caenorhabditis elegans* reference X chromosome (Steinegger and Salzberg, 2020). They also found 114,035 contaminated sequences affecting 2,767 species in RefSeq, and over two million contaminated sequences in GenBank, supporting the recommendation in Issue #5 to avoid the use of NCBI nt (Steinegger and Salzberg, 2020). To the author’s knowledge, NCBI does not perform systematic detection or correction of chimeric sequences in these databases.

Occasionally, chimeric sequences result from inappropriate handling of sequencing data, such as merging contigs together. Merchant, Wood and Salzberg identified chimeric cow sequence in a *Neisseria gonorrhoeae* genome, resulting from this issue (Merchant et al., 2014). Viral reference sequences are particularly prone to chimeric contamination, as they may contain host sequences on the ends if integrated into the host genome. Chen et al. found that 85.5% of contamination cases in viral genomes were of chimeric fragments, and 668 viral sequences in GenBank and NCBI Viral Genome Resources were contaminated (Chen et al., 2022).

Chimeric sequence contamination can be avoided by assessing sequences and their expected taxonomic composition before inclusion into the reference database. Notably, the methods for detecting chimeric sequence contamination are often different from the methods for detecting partitioned sequence contamination. While methods described above generally look at the complete genome assembly, methods for detecting chimeric sequence contamination must examine each contig individually. This assessment is complicated by horizontal gene transfer, a common phenomenon in bacteria, which may appear as regions of a contig originating from different organisms (Orakov et al., 2021). NCBI’s VecScreen can be used to detect vector contamination, and additions have been made to leverage taxonomy to reduce false positives (Schäffer et al., 2018). GUNC uses a database of genes and their expected taxonomic range to assess the phylogenetic homogeneity of the gene content of each contig relative to the entire genome (Orakov et al., 2021). GUNC can therefore flag assemblies where contigs do not have homogeneous gene content. Notably, however, GUNC is limited to prokaryotic genomes and performs better for chimerism across genus or higher ranks (Orakov et al., 2021). CheckV assesses for proviral sequence chimerism with host sequences but is limited to detecting microbial host contamination (Nayfach et al., 2021). Others, such as the authors of KMCP, have used tools like CheckV to mask proviral sequences from the reference database and avoid metagenomic read misclassification (Shen et al., 2023). Other kinds of chimeric sequence contamination can be detected by performing local alignment of sequences across taxa, where low sequence homology is expected. Conterminator performs this alignment with MMSeqs2 (Steinegger and Salzberg, 2020). However, care should be taken as Conterminator only examines interkingdom contamination, as well as cases where the contaminating sequence is less than 20 kilobases and the receptive sequence is more than 20 kilobases, meaning contamination of viral sequences will often be missed. Kraken has also been used to mask parts of contigs which share sequence homology to unexpected taxa (Lu and Salzberg, 2018). As evidenced from the multitude of different approaches, it is likely that multiple approaches are required to accurately detect and mitigate chimeric sequence contamination.

### 2.8 Poor quality reference sequences

Reference sequences may be poor quality due to sequence fragmentation, incompleteness or inaccurate sequence content. Fragmentation is the inclusion of unnaturally split genomic or transcriptomic sequences in the database, while incompleteness is simply missing a portion of the desired genome. Often there is a trade-off between including suboptimal sequences to increase taxonomic representation (Issue #3) while avoiding this issue. This results from the fact that most publicly available genomes were generated with a short-read sequencing platform, and therefore cannot be completed (Segerman, 2020). Incomplete reference sequences manifest similarly to taxonomic underrepresentation (Issue #3) and are therefore not explored further here.

Several aspects of sequence fragmentation hinder metagenomic classification. First, fragmented genomes with short fragments are far more likely to be contaminated (Issues #6 and #7). Breitweiser et al. showed that 99.7% of contaminated contigs and scaffolds in bacterial genomes are shorter than 10 kbp, 99.3% are below 5 kbp, and 92% are below 1 kbp (Breitwieser et al., 2019). Yet, just 0.34% of the total sequence of those assemblies is in scaffolds smaller than 1 kbp, 1.8% of sequence is in scaffolds smaller than 5 kbp, and 3.6% of the total bacterial and archaeal sequence in RefSeq is in contigs that are less than 10 kbp in size (Breitwieser et al., 2019).

However, the issue of fragmentation goes beyond sequence contamination. Query sequences aligning to reference sequence contig ends and inaccurately resolved repeats may be missed in classification (Breitwieser et al., 2019). This is particularly problematic for incomplete genome assemblies which are fragmented due to assembly from short read sequencing data (Aganezov et al., 2022). For most organisms, ribosomal sequences are the most abundant reads in a metagenomic sample; yet their corresponding genomic region in reference genomes is usually highly fragmented, precluding accurate classification of the corresponding reads (Yuan et al., 2015; Chan et al., 2023). Similarly, reads deriving from repeats in the human genome are significantly more likely to contaminate bacterial genomes because they are not identified during an initial filtering/classification step (see Issue #5) (Breitwieser et al., 2019).

Next, many genomes are circular, yet the linear representation of FASTA format necessitates representing the sequence broken at a certain point. While it is generally accepted to break the sequence at *dnaA*, near the origin of replication, many bacterial genomes do not follow this convention, and it is not applicable to plasmids, viruses and other circular sequences (Hunt et al., 2015). Inconsistent circularization may hinder duplicate reference sequence identification if the sequences are rotated differently. When aligning long reads or query contigs against reference sequences, inappropriate or inconsistent breakpoints may also hinder examination and visualization of structural variation.

Looking beyond fragmentation, the accuracy of reference sequences can pose issues for metagenomic classification. Sequence accuracy of reference sequences depends on the purity of the original sample, accuracy of the sequencing technology, sequencing depth and bioinformatic methods used to build an assembly. Ion Torrent sequencing typically suffers from a greater insertion and deletion error rate than other platforms; this manifests as a significantly increased prevalence of pseudogenes and protein frameshifts in reference sequences generated with this platform (Segerman, 2020). Similarly, Oxford Nanopore Technologies (ONT) sequencing has traditionally suffered from high rates of insertion and deletion errors around homopolymers. While the accuracy of the platform has significantly improved with the latest chemistry and basecallers, at least 3% of RefSeq genomes were generated with the error-prone ONT chemistries (Segerman, 2020; Sereika et al., 2022).

The quality of reference sequences can be ensured via tight quality control of included sequences. Fragmented genomes, or short fragments within those genomes, can be excluded from the database. Genome fragmentation can be assessed with tools such as QUAST, which generates assembly statistics, and BUSCO, which examines completeness (or lack thereof) using single copy orthologs (Gurevich et al., 2013; Manni et al., 2021). Some tools, such as MetaPhlAn, exclude incomplete genomes from their databases; however, this may exacerbate taxonomic underrepresentation (Blanco-Míguez et al., 2023). Genomes generated with certain sequencing platforms or bioinformatic approaches known to produce more erroneous assemblies can be excluded to improve sequence content. Furthermore, other metrics for quality control can be established, such as the minimum sequencing depth required, to ensure accurate sequence content.

### 2.9 Low complexity masking

Many organisms contain long stretches of low complexity sequences. These sequences are usually uninformative for metagenomic classification and can result in false positive classifications (Sharon et al., 2005; Camacho et al., 2009; Frith et al., 2010). Masking these sequences usually results in faster, more precise classification (Frith, 2011). However, not all public or popular databases perform low complexity masking. For example, as of July, 2023, the downloadable versions of BLAST and KMCP databases, as well as the default database build of ganon, do not include low complexity masking (Camacho et al., 2009; Piro et al., 2020; Shen et al., 2023).

Low complexity sequence masking of nucleotide sequences can be performed with NCBI’s dustmasker tool (Morgulis et al., 2006). Considerations around low complexity masking include whether the masking is only applied at a seeding stage of sequence alignment, or whether it also prevents an extension stage from including masked regions. In general, masking only the seed stage is sufficient to prevent most spurious alignments, and is the approach taken by NCBI’s online BLASTN tool. Kraken 2, Centrifuge and other metagenomic classifiers also rely on dustmasker or a similar implementation to perform masking (Kim et al., 2016; Wood et al., 2019). While dustmasker has a default threshold to mask, others have shown adjusting the intensity of masking can affect metagenomic classification (Frith, 2011). Care must also be taken when choosing which sequences to mask. For example, it may be counterproductive to mask host sequences, which have a high probability of being truly present in a sample, as reads deriving from these regions may cause false positive alignments to non-host sequences (Gihawi et al., 2023).

### 2.10 Database maintenance and updating

As evidenced by the above nine issues, maintaining reference sequence databases is complex and resource intensive, requiring sustained funding and a team of curators, bioinformaticians, software engineers and microbiologists. With the rapid expansion of sequencing, the identification and reclassification of species has drastically accelerated (Haft et al., 2018; Nasko et al., 2018; Piro et al., 2020). Reference sequence databases even days old may be outdated and produce inaccurate results. Yet, the barriers to generating a reference sequence database have led groups to build a database and rarely or never update it again.

Occasionally, rapid database growth completely precludes generation of new indices based on the computational resources and processing time required to build a newer version of the database. Piro et al. reported that they could not build databases from RefSeq with Krakenuniq or Centrifuge in less than 24 h and with 48 threads, and Plyusnin et al. documented failure to build the Centrifuge NCBI nt database after 70 h and with 32 threads (Piro et al., 2020; Plyusnin et al., 2023). Likely for this reason, the latest available Centrifuge database leveraging NCBI nt as its source was built in 2018, with multiple users requesting a newer compilation of the database (GitHub, 2022).

Even with tools that are built to handle growing reference sequence databases, many tools suffer from “dormant rot,” the inability to install or run the tool after the absence of updates and maintenance (Deschamps et al., 2023). The bioinformatics landscape is particularly rife with examples of poorly maintained and documented tools, as developers are often incentivized to build and publish new tools instead of ensure long term sustainability (Ferenc et al., 2022).

The aforementioned issue can be mitigated with a team approach to database management. Databases and the tools to build them should be updated frequently and periodically. Metagenomic classification tools which can accommodate large databases in their indexing process should ideally be chosen for analysis. Database curation methods should be automated to enable rapid and easy updating of databases, within a version control and quality management system. Emerging infections may necessitate interval update of the database between periodic updates. When databases are updated, continuous integration tests should be run to evaluate the integrity, validity and accuracy of a new database.

## 3 Conclusion

This review highlights ten common issues with reference sequence databases. These issues have significant impact on metagenomic classification accuracy and downstream interpretation, yet the research, clinical and public health communities continue to use databases fraught with these issues. Often this is because most analyses use the default databases of tools such as Kraken 2, Centrifuge, ganon and others; these databases offer the path of least resistance to performing metagenomic analysis: they are easy to compile or download and guaranteed to work with their paired tool (Kim et al., 2016; Wood et al., 2019; Piro et al., 2020). However, the simplicity and ease of using default databases comes at odds with the absence of quality control measures required to ensure accurate classification.

As reviewed here, compiling a high quality reference sequence database to support accurate and meaningful metagenomic classification remains a challenging endeavor. These efforts are particularly valuable in high stakes applications, such as clinical metagenomics, biodefense and public health. Much of this effort can be automated to reduce manual curation; however, significant expertise and resources are still required to set up and perform the automation. Processing millions of sequences through a plethora of tools requires experience with cloud processing, containerization, workflow management and process optimization. Each database design decision, from the inclusion and exclusion of sequences to the filtering of erroneous sequences and processing of retained sequences, requires meticulous thought and assessment. A suite of many tools, including custom tools, is required to handle all the database issues, and tools may interact in unexpected and unintended ways. Even with a robust suite of quality control, manual intervention and curation are typically required for edge cases and outliers. Furthermore, assuming a perfect metagenomic sequencing database, expertise is required to understand metagenomic sequencing results and place them into context. The infamous cases of biothreat agents detected in the New York subway, and more recently, extremophile organisms in the tumor microbiome, highlight these cases (Ackelsberg et al., 2015; Gihawi et al., 2023).

Looking forward, we are excited for several trends in the field to improve reference sequence databases. First, refinements to the accepted taxonomy based on genomics will enable more rational classification of organisms. These changes will in turn enable more systematic evaluation of sequence databases using techniques such as sequence homology and conservation. While it will likely take a long time for some of the classic taxonomic “errors,” such as *E. coli* and *Shigella* spp, to be reclassified, improvements are being made across the microbial spectrum. Recent examples include the abandonment of dual nomenclature for fungi, changes to the *Staphylococcaceae* family and *Lactobacillus* genus, and reclassification of influenza viruses (Lücking et al., 2021; Walker et al., 2021; Munson and Carroll, 2022).

In addition to an accurate taxonomical framework, long read sequencing is set to enable more accurate and complete reference sequences. Recent improvements to Oxford Nanopore and Pacific Biosciences sequencing enables near-perfect bacterial genomes, and effects on metagenome-assembled and eukaryotic genomes will likely be even more striking (Sereika et al., 2022; Yu et al., 2023). Reference sequences generated on these platforms will supplant or improve short-read reference sequences, as in the case of the human reference genome, and mitigate many of the discussed issues in this review (Aganezov et al., 2022). Even with short-read sequencing, newer techniques such as Hi-C and single-cell sequencing have become increasingly popular and driven improvements in reference sequence quality. Both Hi-C sequencing, which cross-links DNA molecules in close physical proximity, and single-cell sequencing, which recovers nucleic acid sequences from individual cells, have enabled high quality reference genomes and plasmid-host linkage from metagenomes and uncultured organisms (Blainey, 2013; Stewart et al., 2018).

Beyond the sequencing, significant efforts are being made at upstream sequence repositories such as NCBI to improve the submission process and flag problematic sequences after submission. RefSeq has continually improved its curation process. However, much work remains on top of upstream curation, as highlighted in this review. Algorithmic and tool improvements, such as graph-based tools, will enable the large-scale comparison of genomes and identification of anomalous sequences. Artificial intelligence advances, such as pattern recognition, will also be of use to automatically identify erroneous or outlier sequences for further inspection.

Together, these technological advancements, along with the established mitigation measures reported here, present a promising path towards better, high quality reference sequence databases. These improvements will continue to expand metagenomic sequencing as a pivotal technology in the understanding of health, disease and our environment.

## References

[B1] Ackelsberg J. Rakeman J. Hughes S. Petersen J. Mead P. Schriefer M. (2015). Lack of evidence for plague or anthrax on the New York city subway. Cell. Syst. 1, 4–5. 10.1016/j.cels.2015.07.008 27135683

[B2] Aganezov S. Yan S. M. Soto D. C. Kirsche M. Zarate S. Avdeyev P. (2022). A complete reference genome improves analysis of human genetic variation. Science 376, eabl3533. eabl3533. 10.1126/science.abl3533 35357935 PMC9336181

[B3] Amos B. Aurrecoechea C. Barba M. Barreto A. Basenko E. Bażant W. (2022). VEuPathDB: the eukaryotic pathogen, vector and host bioinformatics resource center. Nucleic Acids Res. 50, D898–D911. 10.1093/nar/gkab929 34718728 PMC8728164

[B4] Beaz-Hidalgo R. Hossain M. J. Liles M. R. Figueras M. J. (2015). Strategies to avoid wrongly labelled genomes using as example the detected wrong taxonomic affiliation for Aeromonas genomes in the GenBank database. PLOS ONE 10, e0115813. 10.1371/journal.pone.0115813 25607802 PMC4301921

[B5] Blainey P. C. (2013). The future is now: single-cell genomics of bacteria and archaea. FEMS Microbiol. Rev. 37, 407–427. 10.1111/1574-6976.12015 23298390 PMC3878092

[B6] Blanco-Míguez A. Beghini F. Cumbo F. McIver L. J. Thompson K. N. Zolfo M. (2023). Extending and improving metagenomic taxonomic profiling with uncharacterized species using MetaPhlAn 4. Nat. Biotechnol. 41, 1633–1644. 10.1038/s41587-023-01688-w 36823356 PMC10635831

[B7] Bowers R. M. Kyrpides N. C. Stepanauskas R. Harmon-Smith M. Doud D. Reddy T. B. K. (2017). Minimum information about a single amplified genome (MISAG) and a metagenome-assembled genome (MIMAG) of bacteria and archaea. Nat. Biotechnol. 35, 725–731. 10.1038/nbt.3893 28787424 PMC6436528

[B8] Breitwieser F. P. Baker D. N. Salzberg S. L. (2018). KrakenUniq: confident and fast metagenomics classification using unique k-mer counts. Genome Biol. 19, 198–210. 10.1186/s13059-018-1568-0 30445993 PMC6238331

[B9] Breitwieser F. P. Pertea M. Zimin A. V. Salzberg S. L. (2019). Human contamination in bacterial genomes has created thousands of spurious proteins. Genome Res. 29, 954–960. 10.1101/gr.245373.118 31064768 PMC6581058

[B10] Browne P. D. Nielsen T. K. Kot W. Aggerholm A. Gilbert M. T. P. Puetz L. (2020). GC bias affects genomic and metagenomic reconstructions, underrepresenting GC-poor organisms. GigaScience 9, giaa008. 10.1093/gigascience/giaa008 32052832 PMC7016772

[B11] Bush S. J. Connor T. R. Peto T. E. A. Crook D. W. Walker A. S. (2020). Evaluation of methods for detecting human reads in microbial sequencing datasets. Microb. Genom 6, mgen000393. 10.1099/mgen.0.000393 32558637 PMC7478626

[B12] Camacho C. Coulouris G. Avagyan V. Papadopoulos J. Bealer K. (2009). BLAST+: architecture and applications. BMC Bioinforma. 10, 421. 10.1186/1471-2105-10-421 PMC2803857 20003500

[B13] Chan A. P. Siddique A. Desplat Y. Choi Y. Ranganathan S. Choudhary K. S. (2023). A CRISPR-enhanced metagenomic NGS test to improve pandemic preparedness. Cell. Rep. Methods 3, 100463. 10.1016/j.crmeth.2023.100463 37323571 PMC10110940

[B14] Chandrakumar I. Gauthier N. P. G. Nelson C. Bonsall M. B. Locher K. Charles M. (2022). BugSplit enables genome-resolved metagenomics through highly accurate taxonomic binning of metagenomic assemblies. Commun. Biol. 5, 151–210. 10.1038/s42003-022-03114-4 35194141 PMC8864044

[B15] Chen J. Sun Y. Yan X. Ren Z. Wang G. Liu Y. (2022). Elimination of foreign sequences in eukaryotic viral reference genomes improves the accuracy of virome analysis. mSystems 7, e0090722–22. 10.1128/msystems.00907-22 36286492 PMC9765019

[B16] Chen Y.-C. Liu T. Yu C.-H. Chiang T. Y. Hwang C. C. (2013). Effects of GC bias in next-generation-sequencing data on *de novo* genome assembly. PLOS ONE 8, e62856. 10.1371/journal.pone.0062856 23638157 PMC3639258

[B17] Chiu C. Y. Miller S. A. (2019). Clinical metagenomics. Nat. Rev. Genet. 20, 341–355. 10.1038/s41576-019-0113-7 30918369 PMC6858796

[B18] Ciufo S. Kannan S. Sharma S. Badretdin A. Clark K. Turner S. (2018). Using average nucleotide identity to improve taxonomic assignments in prokaryotic genomes at the NCBI. Int. J. Syst. Evol. Microbiol. 68, 2386–2392. 10.1099/ijsem.0.002809 29792589 PMC6978984

[B19] Cornet L. Baurain D. (2022). Contamination detection in genomic data: more is not enough. Genome Biol. 23, 60. 10.1186/s13059-022-02619-9 35189924 PMC8862208

[B20] Cornet L. Meunier L. Vlierberghe M. V. Léonard R. R. Durieu B. Lara Y. (2018). Consensus assessment of the contamination level of publicly available cyanobacterial genomes. PLOS ONE 13, e0200323. 10.1371/journal.pone.0200323 30044797 PMC6059444

[B21] Deschamps J. Dalle Nogare D. Jug F. (2023). Better research software tools to elevate the rate of scientific discovery or why we need to invest in research software engineering. Front. Bioinforma. 3, 1255159. 10.3389/fbinf.2023.1255159 PMC10438982 37600971

[B22] Devanga Ragupathi N. K. Muthuirulandi Sethuvel D. P. Inbanathan F. Y. Veeraraghavan B. (2018). Accurate differentiation of *Escherichia coli* and Shigella serogroups: challenges and strategies. New Microbes New Infect. 21, 58–62. 10.1016/j.nmni.2017.09.003 29204286 PMC5711669

[B23] Fan J. Huang S. Chorlton S. D. (2021). BugSeq: a highly accurate cloud platform for long-read metagenomic analyses. BMC Bioinforma. 22, 160–212. 10.1186/s12859-021-04089-5 PMC7993542 33765910

[B24] Federhen S. (2015). Type material in the NCBI taxonomy database. Nucleic Acids Res. 43, D1086–D1098. 10.1093/nar/gku1127 25398905 PMC4383940

[B25] Ferenc K. Otto K. Neto F. G. de O. Empirical study on software and process quality in bioinformatics tools. 2022; 2022.

[B26] Frith M. C. (2011). Gentle masking of low-complexity sequences improves homology search. PLoS One 6, e28819. 10.1371/journal.pone.0028819 22205972 PMC3242753

[B27] Frith M. C. Hamada M. Horton P. (2010). Parameters for accurate genome alignment. BMC Bioinforma. 11, 80. 10.1186/1471-2105-11-80 PMC2829014 20144198

[B28] Gauthier N. P. G. Chorlton S. D. Krajden M. Manges A. R. (2023). Agnostic sequencing for detection of viral pathogens. Clin. Microbiol. Rev. 36, e0011922–22. 10.1128/cmr.00119-22 36847515 PMC10035330

[B29] Gihawi A. Ge Y. Lu J. Major data analysis errors invalidate cancer microbiome findings. 2023; 2023. 10.1128/mbio.01607-23 PMC10653788 37811944

[B30] Gilbert J. A. Blaser M. J. Caporaso J. G. Jansson J. K. Lynch S. V. Knight R. (2018). Current understanding of the human microbiome. Nat. Med. 24, 392–400. 10.1038/nm.4517 29634682 PMC7043356

[B31] GitHub (2022). Possibility to preapare a new nt database · Issue #227 · DaehwanKimLab/centrifuge. GitHub.

[B32] Gurevich A. Saveliev V. Vyahhi N. Tesler G. (2013). QUAST: quality assessment tool for genome assemblies. Bioinformatics 29, 1072–1075. 10.1093/bioinformatics/btt086 23422339 PMC3624806

[B33] Haft D. H. DiCuccio M. Badretdin A. Brover V. Chetvernin V. O’Neill K. (2018). RefSeq: an update on prokaryotic genome annotation and curation. Nucleic Acids Res. 46, D851–D860. 10.1093/nar/gkx1068 29112715 PMC5753331

[B34] Huang C. Wang Y. Li X. Ren L. Zhao J. Hu Y. (2020). Clinical features of patients infected with 2019 novel coronavirus in Wuhan, China. Lancet 395, 497–506. 10.1016/s0140-6736(20)30183-5 31986264 PMC7159299

[B35] Huang N. Li H. (2023). miniBUSCO: a faster and more accurate reimplementation of BUSCO, 2023. 10.1093/bioinformatics/btad595 PMC10558035 37758247

[B36] Hunt M. Silva N. D. Otto T. D. Parkhill J. Keane J. A. Harris S. R. (2015). Circlator: automated circularization of genome assemblies using long sequencing reads. Genome Biol. 16, 294–310. 10.1186/s13059-015-0849-0 26714481 PMC4699355

[B37] Inzaule S. C. Tessema S. K. Kebede Y. Ogwell Ouma A. E. Nkengasong J. N. (2021). Genomic-informed pathogen surveillance in Africa: opportunities and challenges. Lancet Infect. Dis. 21, e281–e289. 10.1016/s1473-3099(20)30939-7 33587898 PMC7906676

[B38] Janda J. M. Abbott S. L. (2007). 16S rRNA gene sequencing for bacterial identification in the diagnostic laboratory: pluses, perils, and pitfalls. J. Clin. Microbiol. 45, 2761–2764. 10.1128/jcm.01228-07 17626177 PMC2045242

[B39] Johnson S. Parker M. (2020). Ethical challenges in pathogen sequencing: a systematic scoping review. Wellcome Open Res. 5, 119. 10.12688/wellcomeopenres.15806.1 32864469 PMC7445679

[B40] Jorge F. Brealey J. C. Brindley P. J. Buysse M. Cantacessi C. Duron O. (2022). MIxS-SA: a MIxS extension defining the minimum information standard for sequence data from symbiont-associated micro-organisms. ISME Commun. 2, 9–5. 10.1038/s43705-022-00092-w 37938691 PMC9723553

[B41] Kalantar K. L. Carvalho T. de Bourcy C. F. A. Dimitrov B. Dingle G. Egger R. (2020). IDseq—an open source cloud-based pipeline and analysis service for metagenomic pathogen detection and monitoring. GigaScience 9, giaa111. 10.1093/gigascience/giaa111 33057676 PMC7566497

[B42] Kannan S. Sharma S. Ciufo S. Clark K. Turner S. Kitts P. A. (2023). Collection and curation of prokaryotic genome assemblies from type strains at NCBI. Int. J. Syst. Evol. Microbiol. 73, 005707. 10.1099/ijsem.0.005707 36748495 PMC10228379

[B43] Karasikov M. Mustafa H. Danciu D. MetaGraph: indexing and analysing nucleotide archives at petabase-scale. 2020; 2020.

[B44] Kim D. Song L. Breitwieser F. P. Salzberg S. L. (2016). Centrifuge: rapid and sensitive classification of metagenomic sequences. Genome Res. 26, 1721–1729. 10.1101/gr.210641.116 27852649 PMC5131823

[B45] Kuhn J. H. Bào Y. Bavari S. Becker S. Bradfute S. Brauburger K. (2014). Virus nomenclature below the species level: a standardized nomenclature for filovirus strains and variants rescued from cDNA. Arch. Virol. 159, 1229–1237. 10.1007/s00705-013-1877-2 24190508 PMC4010566

[B46] Ladner J. T. Beitzel B. Chain P. S. G. Davenport M. G. Donaldson E. Frieman M. (2014). Standards for sequencing viral genomes in the era of high-throughput sequencing. mBio 5, e01360. 10.1128/mbio.01360-14 24939889 PMC4068259

[B47] Lamperti E. D. Kittelberger J. M. Smith T. F. VillaKomaroff L. (1992). Corruption of genomic databases with anomalous sequence. Nucleic Acids Res. 20, 2741–2747. 10.1093/nar/20.11.2741 1614861 PMC336916

[B48] Li H. (2018). Minimap2: pairwise alignment for nucleotide sequences. Bioinformatics 34, 3094–3100. 10.1093/bioinformatics/bty191 29750242 PMC6137996

[B49] Lu J. Salzberg S. L. (2018). Removing contaminants from databases of draft genomes. PLOS Comput. Biol. 14, e1006277. 10.1371/journal.pcbi.1006277 29939994 PMC6034898

[B50] Lücking R. Aime M. C. Robbertse B. Miller A. N. Aoki T. Ariyawansa H. A. (2021). Fungal taxonomy and sequence-based nomenclature. Nat. Microbiol. 6, 540–548. 10.1038/s41564-021-00888-x 33903746 PMC10116568

[B51] Lupo V. Van Vlierberghe M. Vanderschuren H. Kerff F. Baurain D. Cornet L. (2021). Contamination in reference sequence databases: time for divide-and-rule tactics. Front. Microbiol. 12, 755101. 10.3389/fmicb.2021.755101 34745061 PMC8570097

[B52] Manni M. Berkeley M. R. Seppey M. Simão F. A. Zdobnov E. M. (2021). BUSCO update: novel and streamlined workflows along with broader and deeper phylogenetic coverage for scoring of eukaryotic, prokaryotic, and viral genomes. Mol. Biol. Evol. 38, 4647–4654. 10.1093/molbev/msab199 34320186 PMC8476166

[B53] Marcelino V R. Holmes E. C. Sorrell T. C. (2020). The use of taxon-specific reference databases compromises metagenomic classification. BMC Genomics 21, 1–5. 10.1186/s12864-020-6592-2 PMC7045516 32106809

[B54] Merchant S. Wood D. E. Salzberg S. L. (2014). Unexpected cross-species contamination in genome sequencing projects. PeerJ 2, e675. 10.7717/peerj.675 25426337 PMC4243333

[B55] Méric G. Wick R. R. Watts S. C. Correcting index databases improves metagenomic studies. 2019; 712166

[B56] Morgulis A. Gertz E. M. Schäffer A. A. Agarwala R. (2006). A fast and symmetric DUST implementation to mask low-complexity DNA sequences. J. Comput. Biol. 13, 1028–1040. 10.1089/cmb.2006.13.1028 16796549

[B57] Muñoz-Escalante J. C. Comas-García A. Bernal-Silva S. Robles-Espinoza C. D. Gómez-Leal G. Noyola D. E. (2019). Respiratory syncytial virus A genotype classification based on systematic intergenotypic and intragenotypic sequence analysis. Sci. Rep. 9, 20097. 10.1038/s41598-019-56552-2 31882808 PMC6934736

[B58] Munson E. Carroll K. C. (2022). Summary of novel bacterial isolates derived from human clinical specimens and nomenclature revisions published in 2018 and 2019. J. Clin. Microbiol. 61, e01309-20–e01322. 10.1128/JCM.01309-20 PMC8111135 32967902

[B59] Naccache S. N. Federman S. Veeraraghavan N. Zaharia M. Lee D. Samayoa E. (2014). A cloud-compatible bioinformatics pipeline for ultrarapid pathogen identification from next-generation sequencing of clinical samples. Genome Res. 24, 1180–1192. 10.1101/gr.171934.113 24899342 PMC4079973

[B60] Nasko D. J. Koren S. Phillippy A. M. Treangen T. J. (2018). RefSeq database growth influences the accuracy of k-mer-based lowest common ancestor species identification. Genome Biol. 19, 165–210. 10.1186/s13059-018-1554-6 30373669 PMC6206640

[B61] Nayfach S. Camargo A. P. Schulz F. Eloe-Fadrosh E. Roux S. Kyrpides N. C. (2021). CheckV assesses the quality and completeness of metagenome-assembled viral genomes. Nat. Biotechnol. 39, 578–585. 10.1038/s41587-020-00774-7 33349699 PMC8116208

[B62] Oh J. Byrd A. L. Park M. Kong H. Segre J. (2016). Temporal stability of the human skin microbiome. Cell. 165, 854–866. 10.1016/j.cell.2016.04.008 27153496 PMC4860256

[B63] O Leary N. A. Wright M. W. Brister J. R. Ciufo S. Haddad D. McVeigh R. (2016). Reference sequence (RefSeq) database at NCBI: current status, taxonomic expansion, and functional annotation. Nucleic Acids Res. 44, D733–D745. 10.1093/nar/gkv1189 26553804 PMC4702849

[B64] Orakov A. Fullam A. Coelho L. P. Khedkar S. Szklarczyk D. Mende D. R. (2021). GUNC: detection of chimerism and contamination in prokaryotic genomes. Genome Biol. 22, 178. 10.1186/s13059-021-02393-0 34120611 PMC8201837

[B65] Park S.-C. Lee K. Kim Y. O. Won S. Chun J. (2019). Large-scale genomics reveals the genetic characteristics of seven species and importance of phylogenetic distance for estimating pan-genome size. Front. Microbiol. 10, 834. 10.3389/fmicb.2019.00834 31068915 PMC6491781

[B66] Parks D. H. Chuvochina M. Chaumeil P.-A. Rinke C. Mussig A. J. Hugenholtz P. (2020). A complete domain-to-species taxonomy for Bacteria and Archaea. Nat. Biotechnol. 38, 1079–1086. 10.1038/s41587-020-0501-8 32341564

[B67] Parks D. H. Chuvochina M. Rinke C. Mussig A. J. Chaumeil P. A. Hugenholtz P. (2022). GTDB: an ongoing census of bacterial and archaeal diversity through a phylogenetically consistent, rank normalized and complete genome-based taxonomy. Nucleic Acids Res. 50, D785–D794. 10.1093/nar/gkab776 34520557 PMC8728215

[B68] Parks D. H. Imelfort M. Skennerton C. T. Hugenholtz P. Tyson G. W. (2015). CheckM: assessing the quality of microbial genomes recovered from isolates, single cells, and metagenomes. Genome Res. 25, 1043–1055. 10.1101/gr.186072.114 25977477 PMC4484387

[B69] Patel R. (2015). MALDI-TOF MS for the diagnosis of infectious diseases. Clin. Chem. 61, 100–111. 10.1373/clinchem.2014.221770 25278500

[B70] Piro V. C. Dadi T. H. Seiler E. Reinert K. Renard B. Y. (2020). ganon: precise metagenomics classification against large and up-to-date sets of reference sequences. Bioinformatics 36, i12–i20. i12–i20. 10.1093/bioinformatics/btaa458 32657362 PMC7355301

[B71] Plyusnin I. Vapalahti O. Sironen T. Kant R. Smura T. (2023). Enhanced viral metagenomics with lazypipe 2. Viruses 15, 431. 10.3390/v15020431 36851645 PMC9960287

[B72] Quast C. Pruesse E. Yilmaz P. Gerken J. Schweer T. Yarza P. (2013). The SILVA ribosomal RNA gene database project: improved data processing and web-based tools. Nucleic Acids Res. 41, D590–D596. 10.1093/nar/gks1219 23193283 PMC3531112

[B73] Rachtman E. Bafna V. Mirarab S. (2021). CONSULT: accurate contamination removal using locality-sensitive hashing. NAR Genomics Bioinforma. 3, lqab071. 10.1093/nargab/lqab071 PMC8340999 34377979

[B74] Ramaekers K. Rector A. Cuypers L. Lemey P. Keyaerts E. Van Ranst M. (2020). Towards a unified classification for human respiratory syncytial virus genotypes. Virus Evol. 6, veaa052. 10.1093/ve/veaa052 33072402 PMC7552823

[B75] Robertson J. Bessonov K. Schonfeld J. Nash J. H. E. (2020). Universal whole-sequence-based plasmid typing and its utility to prediction of host range and epidemiological surveillance. Microb. Genomics 6, e000435. 10.1099/mgen.0.000435 PMC7660255 32969786

[B76] Roux S. Adriaenssens E. M. Dutilh B. E. Koonin E. V. Kropinski A. M. Krupovic M. (2019). Minimum information about an uncultivated virus genome (MIUViG). Nat. Biotechnol. 37, 29–37. 10.1038/nbt.4306 30556814 PMC6871006

[B77] Rumbavicius I. Rounge T. B. Rognes T. (2023). HoCoRT: host contamination removal tool. BMC Bioinforma. 24, 371–378. 10.1186/s12859-023-05492-w PMC10544359 37784008

[B78] Saary P. Mitchell A. L. Finn R. D. (2020). Estimating the quality of eukaryotic genomes recovered from metagenomic analysis with EukCC. Genome Biol. 21, 244–321. 10.1186/s13059-020-02155-4 32912302 PMC7488429

[B79] Schäffer A. A. Nawrocki E. P. Choi Y. Kitts P. A. Karsch-Mizrachi I. McVeigh R. (2018). VecScreen_plus_taxonomy: imposing a tax(onomy) increase on vector contamination screening. Bioinformatics 34, 755–759. 10.1093/bioinformatics/btx669 29069347 PMC6030928

[B80] Schneider V. A. Graves-Lindsay T. Howe K. Bouk N. Chen H. C. Kitts P. A. (2017). Evaluation of GRCh38 and *de novo* haploid genome assemblies demonstrates the enduring quality of the reference assembly. Genome Res. 27, 849–864. 10.1101/gr.213611.116 28396521 PMC5411779

[B81] Schoch C. L. Ciufo S. Domrachev M. Hotton C. L. Kannan S. Khovanskaya R. (2020). NCBI Taxonomy: a comprehensive update on curation, resources and tools. Database 2020, baaa062. 10.1093/database/baaa062 32761142 PMC7408187

[B82] Segerman B. (2020). The most frequently used sequencing Technologies and assembly methods in different time segments of the bacterial surveillance and RefSeq genome databases. Front. Cell. Infect. Microbiol. 10, 527102. 10.3389/fcimb.2020.527102 33194784 PMC7604302

[B83] Sereika M. Kirkegaard R. H. Karst S. M. Michaelsen T. Y. Sørensen E. A. Wollenberg R. D. (2022). Oxford Nanopore R10.4 long-read sequencing enables the generation of near-finished bacterial genomes from pure cultures and metagenomes without short-read or reference polishing. Nat. Methods 19, 823–826. 10.1038/s41592-022-01539-7 35789207 PMC9262707

[B84] Sharon I. Birkland A. Chang K. El-Yaniv R. Yona G. (2005). Correcting BLAST e-values for low-complexity segments. J. Comput. Biol. 12, 980–1003. 10.1089/cmb.2005.12.980 16201917

[B85] Shen W. Xiang H. Huang T. Tang H. Peng M. Cai D. (2023). KMCP: accurate metagenomic profiling of both prokaryotic and viral populations by pseudo-mapping. Bioinformatics 39, btac845. 10.1093/bioinformatics/btac845 36579886 PMC9828150

[B86] Sichtig H. Minogue T. Yan Y. Stefan C. Hall A. Tallon L. (2019). FDA-ARGOS is a database with public quality-controlled reference genomes for diagnostic use and regulatory science. Nat. Commun. 10, 3313. 10.1038/s41467-019-11306-6 31346170 PMC6658474

[B87] Steinegger M. Salzberg S. L. (2020). Terminating contamination: large-scale search identifies more than 2,000,000 contaminated entries in GenBank. Genome Biol. 21, 115. 10.1186/s13059-020-02023-1 32398145 PMC7218494

[B88] Steinegger M. Söding J. (2018). Clustering huge protein sequence sets in linear time. Nat. Commun. 9, 2542. 10.1038/s41467-018-04964-5 29959318 PMC6026198

[B89] Stewart R. D. Auffret M. D. Warr A. Wiser A. H. Press M. O. Langford K. W. (2018). Assembly of 913 microbial genomes from metagenomic sequencing of the cow rumen. Nat. Commun. 9, 870. 10.1038/s41467-018-03317-6 29491419 PMC5830445

[B90] Sunagawa S. Mende D. R. Zeller G. Izquierdo-Carrasco F. Berger S. A. Kultima J. R. (2013). Metagenomic species profiling using universal phylogenetic marker genes. Nat. Methods 10, 1196–1199. 10.1038/nmeth.2693 24141494

[B91] Tomofuji Y. Sonehara K. Kishikawa T. Maeda Y. Ogawa K. Kawabata S. (2023). Reconstruction of the personal information from human genome reads in gut metagenome sequencing data. Nat. Microbiol. 8, 1079–1094. 10.1038/s41564-023-01381-3 37188815 PMC10234815

[B92] Tringe S. G. Rubin E. M. (2005). Metagenomics: DNA sequencing of environmental samples. Nat. Rev. Genet. 6, 805–814. 10.1038/nrg1709 16304596

[B93] Vries L. de Koopmans M. Morton A. van Baal P. (2021). The economics of improving global infectious disease surveillance. BMJ Glob. Health 6, e006597. 10.1136/bmjgh-2021-006597 PMC8413876 34475025

[B94] Walker P. J. Siddell S. G. Lefkowitz E. J. Mushegian A. R. Adriaenssens E. M. Alfenas-Zerbini P. (2021). Changes to virus taxonomy and to the international code of virus classification and nomenclature ratified by the international committee on taxonomy of viruses (2021). Arch. Virol. 166, 2633–2648. 10.1007/s00705-021-05156-1 34231026

[B95] Wick R. R. Judd L. M. Holt K. E. (2018). Deepbinner: demultiplexing barcoded Oxford Nanopore reads with deep convolutional neural networks. PLOS Comput. Biol. 14, e1006583. 10.1371/journal.pcbi.1006583 30458005 PMC6245502

[B96] Wood D. E. Lu J. Langmead B. (2019). Improved metagenomic analysis with Kraken 2. Genome Biol. 20, 257–313. 10.1186/s13059-019-1891-0 31779668 PMC6883579

[B97] Wright R. J. Comeau A. M. Langille M. G. I. (2023). From defaults to databases: parameter and database choice dramatically impact the performance of metagenomic taxonomic classification tools. Microb. Genom 9, mgen000949. 10.1099/mgen.0.000949 36867161 PMC10132073

[B98] Xu Y. Lewandowski K. Lumley S. Pullan S. Vipond R. Carroll M. (2018). Detection of viral pathogens with multiplex Nanopore MinION sequencing: Be careful with cross-talk. Front. Microbiol. 9, 2225. 10.3389/fmicb.2018.02225 30283430 PMC6156371

[B99] Ye S. H. Siddle K. J. Park D. J. Sabeti P. C. (2019). Benchmarking metagenomics tools for taxonomic classification. Cell. 178, 779–794. 10.1016/j.cell.2019.07.010 31398336 PMC6716367

[B100] Yu J.-M. Fu Y.-H. Peng X.-L. Zheng Y. P. He J. S. (2021). Genetic diversity and molecular evolution of human respiratory syncytial virus A and B. Sci. Rep. 11, 12941. 10.1038/s41598-021-92435-1 34155268 PMC8217232

[B101] Yu W. Luo H. Yang J. (2023). Comprehensive Assessment of Eleven de novo HiFi Assemblers on Complex Eukaryotic Genomes and Metagenomes, 2023. 10.1101/gr.278232.123 PMC10984382 38428994

[B102] Yuan C. Lei J. Cole J. Sun Y. (2015). Reconstructing 16S rRNA genes in metagenomic data. Bioinformatics 31, i35–i43. i35–i43. 10.1093/bioinformatics/btv231 26072503 PMC4765874

[B103] Zaharia M. Bolosky W. J. Curtis K. (2011) Faster and more accurate sequence alignment with SNAP. arXiv.org 2011.

[B104] Zhao S. Zhang B. (2015). A comprehensive evaluation of ensembl, RefSeq, and UCSC annotations in the context of RNA-seq read mapping and gene quantification. BMC Genomics 16, 97–14. 10.1186/s12864-015-1308-8 25765860 PMC4339237

[B105] Zhou W. Gay N. Oh J. (2018). ReprDB and panDB: minimalist databases with maximal microbial representation. Microbiome 6, 15. 10.1186/s40168-018-0399-2 29347966 PMC5774170

